# Complete root coverage in severe gingival recession with unfavorable prognosis using the tunneling technique

**DOI:** 10.34172/japid.2020.009

**Published:** 2019-10-24

**Authors:** Mohammad Ahmad Javaid, Aamna Sohail, Raafay Ahmed

**Affiliations:** ^1^Clinical Assistant Professor Graduate Periodontics University of Alberta, Canada; ^2^Department of Cardiology, Sialkot Medical Complex, Pakistan; ^3^University of Washington, USA

**Keywords:** Periodontics, Dentine Hypersensitivity, Gingival Recession, Dental Esthetics

## Abstract

Gingival recession defined as the apical migration of the gingival margin leads to the exposure of root surface. This in turn may lead to compromised esthetics, dentine hypersensitivity and attachment loss. Severe gingival recession is typically managed surgically. However, achieving complete root coverage in cases of severe gingival recession, especially in the mandibular canine region is quite challenging. Different surgical techniques have been described in the literature to manage this condition. Tunnelling technique is one such technique which has shown promising results.Use of connective tissue graft with tunnelling technique has demonstrated favorable results in cases with mild to moderate gingival recession. Here we report a case where connective tissue graft was used in conjunction with tunnelling technique to achieve complete root coverage despite severe gingival recession and unfavorable prognosis.

## Introduction

Gingival recession is a periodontal condition characterized by the apical migration of the gingival margin, exposing the root surface. Gingival recession might lead to compromised esthetics, dentin sensitivity, root caries, increased risk of further recession, attachment loss, and plaque retention.^1,2^

Gingival recession is typically managed by surgical intervention. However, the surgical treatment of gingival recession is challenging and technique-sensitive. The American Academy of Periodontology states that the mean root coverage of gingival recession varies from 67% to 86%, depending on the surgical technique and prognosis of the gingival recession defect.^3^ The prognosis of the surgical outcome of gingival recession depends on the initial classification of the defect.^4^ The very first classification of gingival recession was introduced by Sullivan and Atkins in 1968. Based on this system, gingival recession was defined as 1) deep wide, 2) shallow wide, 3) deep narrow, and 4) shallow narrow. The deep, wide gingival recession had the worst prognosis.^5^ Mlinek further refined the Sullivan and Atkins classification by defining the terms shallow, narrow, wide, and deep. Based on Mlinek’s modification, gingival recession was classified as deep if its height was more than 3 mm. Similarly, gingival recession was classified as wide if the horizontal dimension of the recession was >3 mm. Deep, wide recessions were considered complex and deemed to have the worst prognosis.^6^ Bengue et al^7^ classified gingival recession as U type, V type, and I type. U type defects had the worst prognosis. However, the most universally used classification is Miller’s classification of gingival recession. It categorizes gingival recession into four categories. Class I includes those defects where the marginal tissues do not recede up to the mucogingival junction. In Class II defects, recession extends up to or beyond the mucogingival junction. Class III involves the loss of interdental tissues, including soft tissue and interproximal bone, and Class IV entails severe interdental tissue loss and/or severe tooth malposition.^8^

Other factors suggested in the literature that might influence complete root coverage are positioning of the tooth in the arch, the thickness of the gingival biotype, the presence or absence of keratinized tissue, and situations where a tooth is located out of alveolar housing.^3^ Zucchelli et al^9^ showed the effect of tooth position on complete root coverage. Generally, the percentage of complete root coverage was higher in the anterior teeth as compared to the posterior teeth but the lowest for mandibular canines. Arcoa et al^10^ showed that maxillary teeth are more likely to achieve complete root coverage than mandibular teeth. A recent clinical trial demonstrated that complete root coverage is less likely in cases with thin biotype than in thick biotype.^11^ Others have shown similar results, highlighting the importance of thick gingival biotype.^12^ Similarly, positioning of the tooth outside the alveolar housing has also been implicated with gingival recession.^13^

Different surgical techniques, including laterally positioned flap,^14^ Zucchelli technique,^15^ coronally advanced flap,^16^ and double papillae technique,^17^ have been described in the literature to treat gingival recession.^18^ Furthermore, the use of connective tissue graft, donor tissue such as Alloderm (acellular human tissue matrix derived from cadaveric tissue) and Emdogain (gel containing enamel matrix derivates) has also been suggested.^19^ Among the plethora of techniques, the tunneling technique has the advantage of blood supply from the overlying flap and underlying periosteal bed without compromise in vascularity due to dissection of papillae.^20^ The use of the tunneling technique in cases of severe gingival recession has been a challenge. In the following case report, we describe how the tunneling technique with connective tissue graft can be used to treat difficult cases of severe gingival recession.

## Case Report

**Figure 1:**
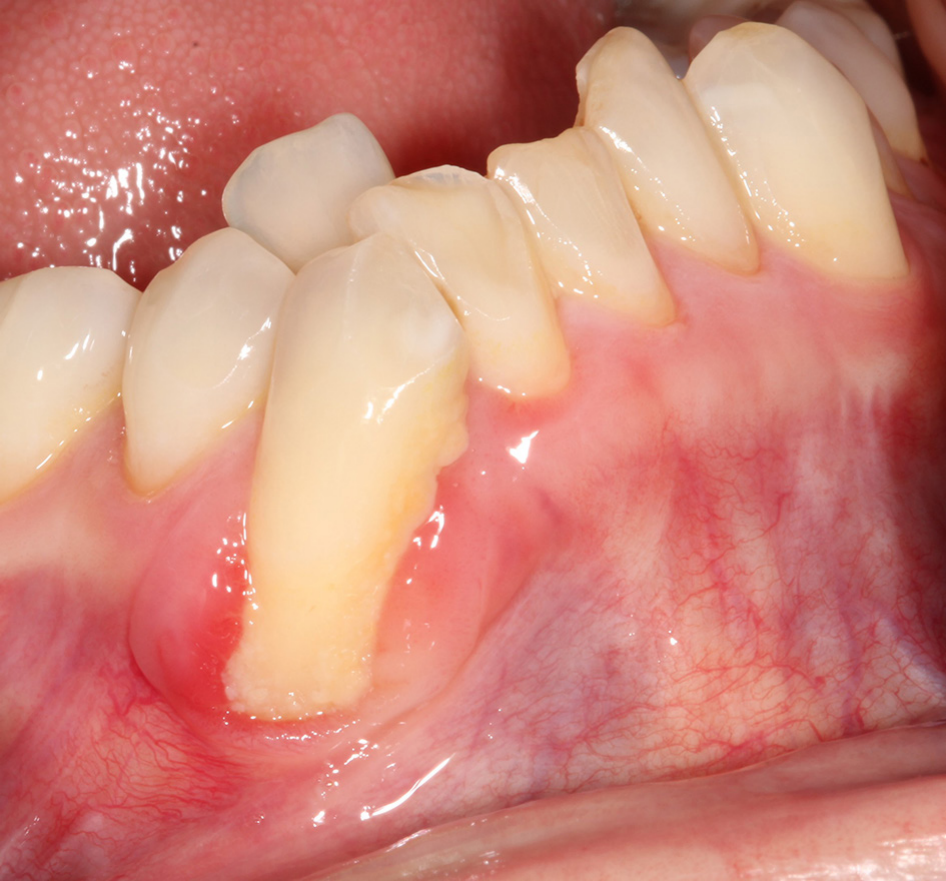
At the time of initial consultation, prior to periodontal therapy.

A 42-year-old Caucasian female with no significant medical history was seen at our periodontal practice for severe dental hypersensitivity at tooth #43 and extraction of nonrestorable tooth #42 (Figure 1 – at the time of initial consultation). The patient was referred from a distant area. This limited our capacity to follow up the patient for a longer period. At the initial examination, orthodontic and prosthodontic consultation was recommended to improve malocclusion and bring tooth #43 inside the alveolar housing for rehabilitation and restoration of the occlusal wear. However, the patient refused orthodontic and occlusal therapy. Written and verbal informed consent was given by the patient before the commencement of the treatment. Periodontal parameters, including probing depth, the height and width of gingival recession, bleeding on probing, the presence or absence of keratinized tissue, frenum pull, vestibular depth, mobility, and position of the tooth with regards to alveolar housing were recorded. Radiographic examination revealed early bone loss. Based on the preliminary examination, tooth #43 was classified as Miller Class III gingival recession (5 mm in height and 5 mm in width as measured by a periodontal probe). The recession was U-shaped. Reduced vestibular depth, thin biotype, and lack of keratinized tissue were noted. The tooth #43 was located out of alveolar housing. Probing depth, mobility, and bleeding on probing were all within the normal limits. Severe gingival recession (height and width of 5 mm), lack of keratinized tissue, thin biotype, shallow vestibular depth, the tooth position, the shape of the defect (U-shaped), and out-of-alveolar housing of the tooth made it a complicated case. Therefore, the patient was informed that complete root coverage was not very likely.

Different treatment options were discussed. After a detailed discussion, the patient wanted to proceed with the extraction of tooth #42 and connective tissue graft at tooth #43. Phase I was comprised of scaling and root planing (Gracey curettes ½ and ¾, and Cavitron were used for mechanical debridement). Eight weeks later, phase II, comprising of extraction of tooth #42 and root coverage of tooth #43, was carried out. After informed consent, profound local anesthesia was obtained, and tooth #42 was extracted using elevators.

The use of coronally advanced flap technique was ruled out due to the lack of keratinized tissue, thin biotype, and the risk of apical migration of the flap with graft exposure. The tunneling technique with connective tissue graft was selected for the management of gingival recession at tooth #43. The tunneling technique has been described in detail elsewhere.^13,21^ Briefly, the Nordland micro-blade was used for infraclavicular incision extending from tooth #44 to tooth #41. The incision line was retraced by the Orban knife. This allowed detachment of the overlying tunnel to the underlying periodontal tissues. The tunnel was further released and undermined using Gracey curette 13/14, and a double-ended periotome. No external incisions were given. The tissues were thoroughly released to the point that flap could be coronally advanced beyond the cementoenamel junction. Careful preparation of the tunnel avoided perforation of the overlying flap. Once adequate periosteal release was obtained, CTG was harvested from the right palatal half using the single-incision technique. Briefly, a single incision extending from the mesial aspect of the first right premolar to the distal aspect of the first molar was made using a 15C blade. A partial-thickness flap was raised using the same blade. The underlying CTG was released, and the overlying flap was sutured using chromic gut 4.0. Hemostasis was achieved. An effort was made to ensure that the CTG was >2 mm in thickness. The harvested CTG was then placed inside the tunnel and secured with the overlying flap via sling sutures using a 6.0 polypropylene suture. Postoperative medications were prescribed, including Ibuprofen 600 mg tid for 5–7 days and 0.12% chlorhexidine mouthwash rinse twice a day for two weeks. The patient was given detailed postoperative instructions and scheduled for follow-up visits. Healing was uneventful. After three weeks, the sutures were removed. The patient was recalled at five weeks to assess the healing process. At this stage, complete root coverage with the elimination of dental hypersensitivity and gain in the attached gingiva was noted. The thickness of gingival tissues and an increase in vestibular depth were also observed. Given the 3-hour one-way drive, the patient requested that further follow-up be done in person only in case of emergency or re-appearance of the gingival recession or dentin hypersensitivity. Three months later, the patient was contacted to assess the healing outcome. The patient reported no sensitivity and was incredibly pleased with the healing outcome.

**Figure 2:**
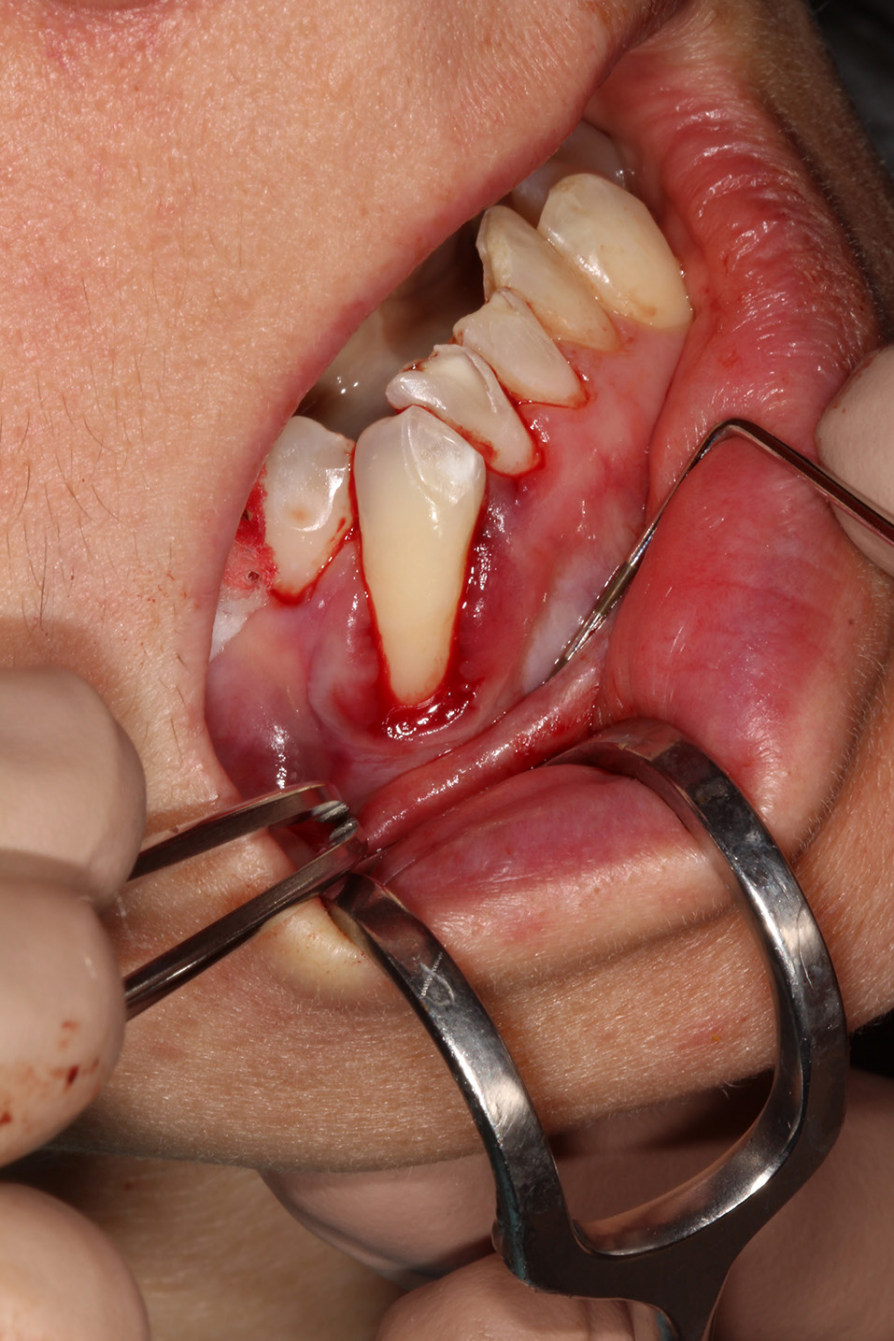
Final tunnel preparation prior to the placement of CTG The harvested CTG positioned on the exposed root surface to check its dimensions prior to placement.

**Figure 3:**
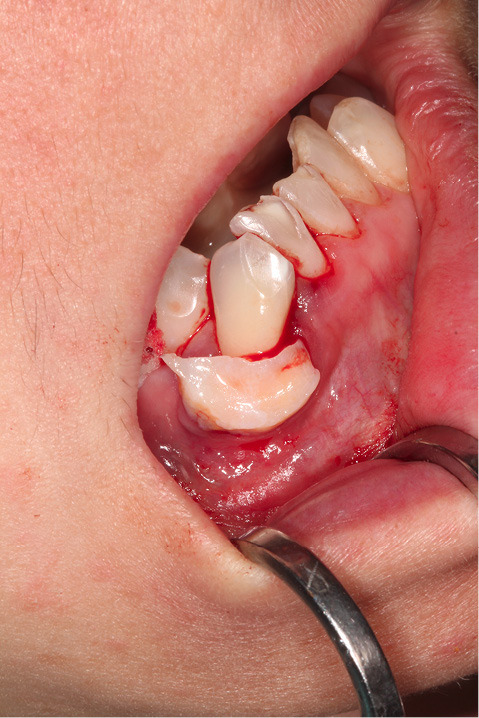
The harvested CTG positioned on the exposed root surface to check its dimensions prior to placement inside the tunnel on the buccal aspect of tooth 43.

**Figure 4:**
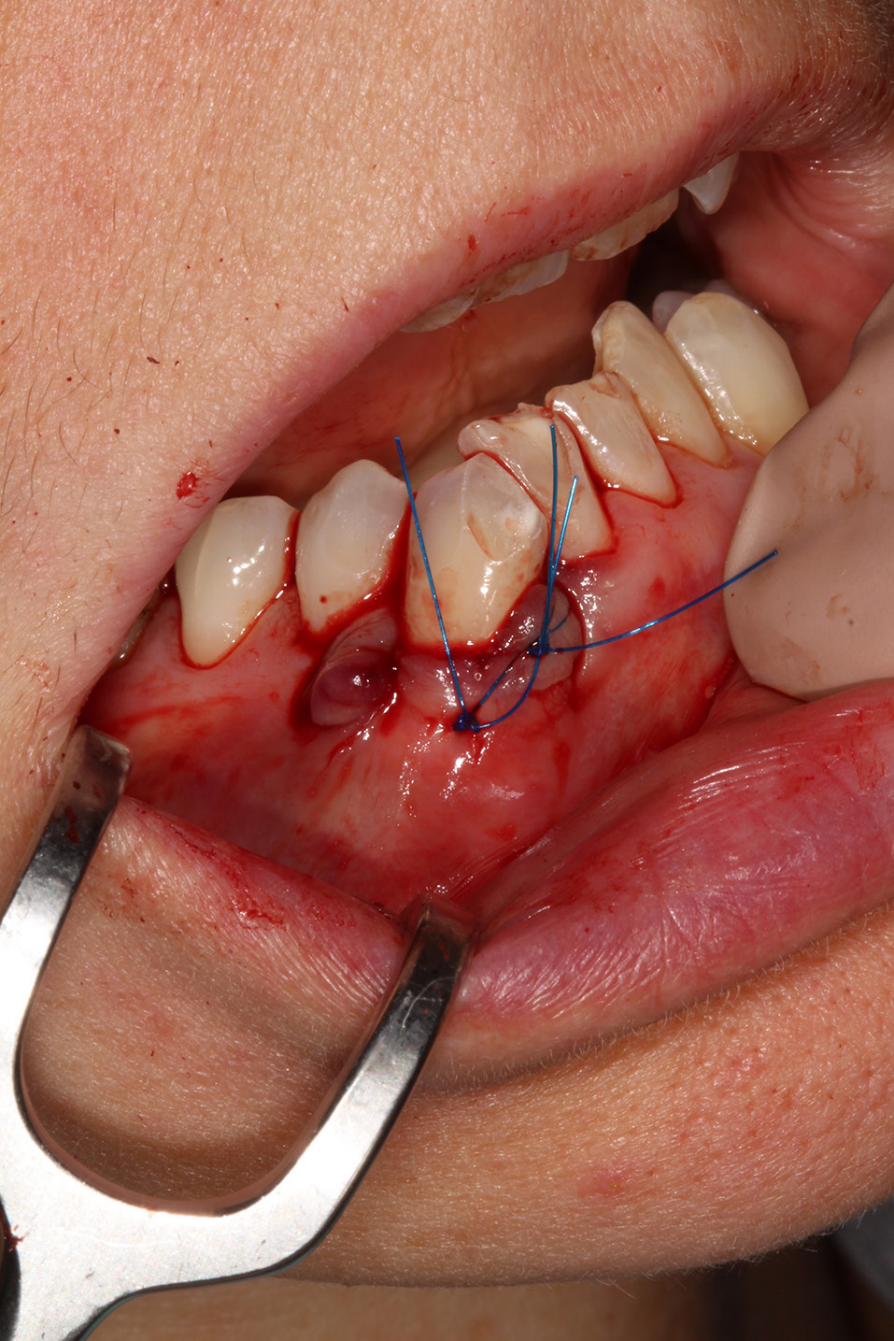
Suturing after the CTG has been positioned inside the tunnel. Please note the sutures were kept longer to prevent poking of the suture ends into the lower lip and buccal mucosa.

**Figure 5:**
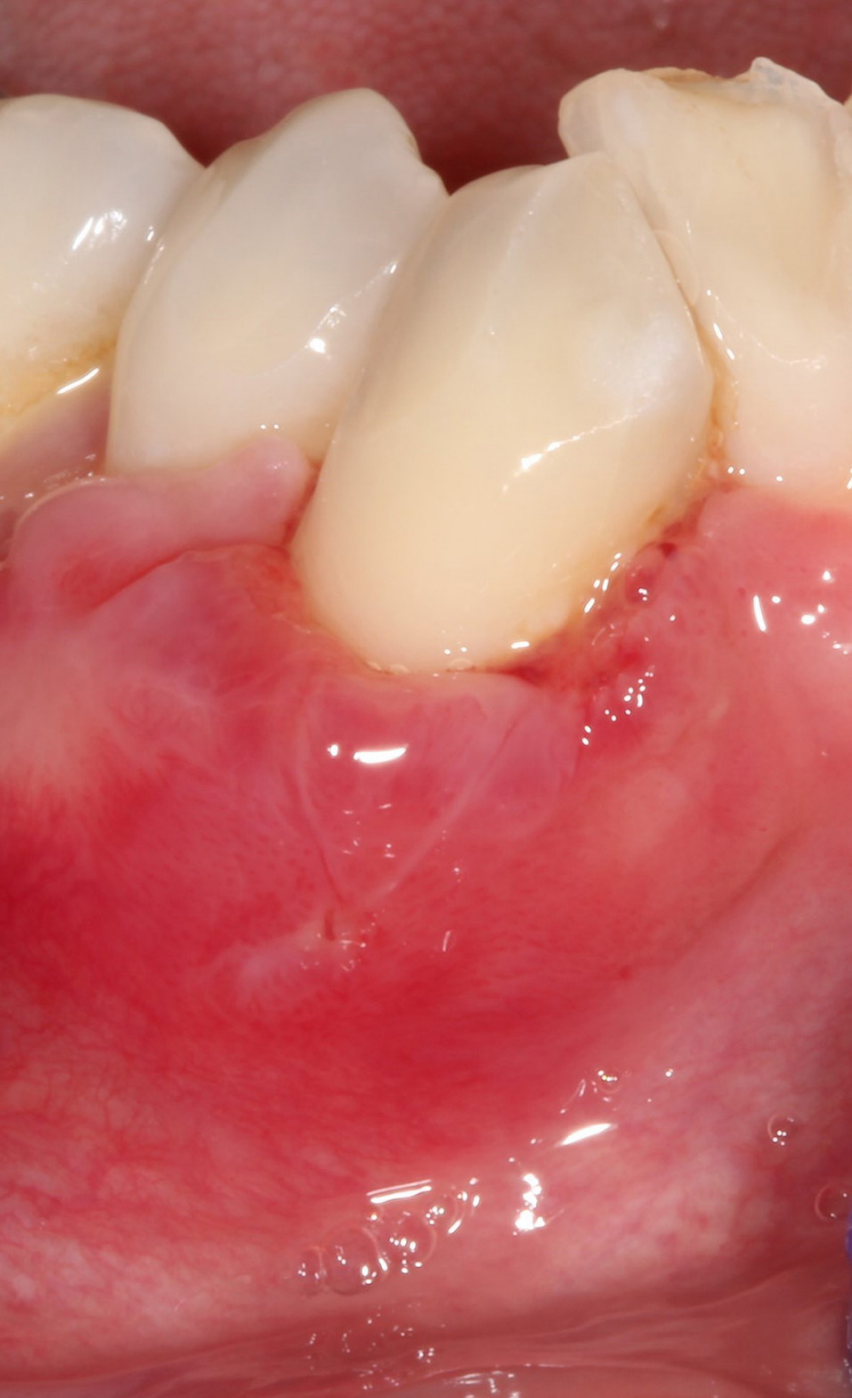
The post op at 5 weeks.

Figure 2 shows the final tunnel preparation before the placement of CTG. Figure 3 shows the harvested CTG positioned on the exposed root surface to check its dimensions before placement inside the tunnel on the buccal aspect of tooth #43. Figure 4 shows suturing after the CTG has been positioned inside the tunnel. Please note that the sutures were kept longer than usual to prevent the poking of the suture ends into the lower lip and buccal mucosa. Figure 5 shows the postoperative condition at five-week interval.

## Discussion

Achieving complete root coverage, especially in the mandibular canine region, is technique-sensitive and difficult.^9^ Factors such as thin biotype, lack of keratinized tissue, shallow vestibule, increased width and height of the recession, and location of the tooth out of alveolar housing can further complicate the case making complete root coverage unlikely. Here, we report a case where all the factors above made it quite unlikely to achieve root coverage.

Several periodontal techniques for root coverage have been described in the literature.^20^ The selection of an appropriate surgical technique in a given case is critical for success. In this case, we decided to use the tunneling technique. The advantage of the tunneling technique is that it avoids vertical incisions and the release of papillae. This not only maximizes the blood supply to the healing CTG but also reduces the risk of graft exposure due to flap contraction or apical migration of the overlying flap. This is critical for achieving optimal results as compromised blood supply, or apical migration of the flap, can result in incomplete root coverage, which in this case would have lead to not only unaesthetic outcome but also persistent dentin sensitivity. Another critical factor for achieving root coverage, regardless of the surgical technique, is the importance of a thorough release of the flap. Finally, there is some evidence to support that thicker graft might result in an increased thickness of biotype and greater root coverage.^3^

A significant limitation of our study, as with any case report, is the sample size. Although case reports, along with professional opinion, only form the base in the hierarchy of evidence-based practice, they do provide the proof of principle and help steer clinicians towards some clinical guidelines in rare or particularly challenging cases. This is evident in our study as we report a case where the severe gingival recession was further complicated by the shape of the defect (U-shaped), height and width of recession (5 mm), thin biotype, lack of keratinized tissue, shallow vestibular depth and location of the tooth out of alveolar housing. Another limitation of the study is the short follow-up of five weeks and follow-up by phone call at three months. Ideally, we would have liked to follow the patient for at least six months. However, given the vast Canadian expanse and a limited number of specialists, we often see patients who are referred from distant areas. That is why, despite our best efforts, it is not always possible to have the patient come back for routine check-ups or long-term follow-ups. This implies that one can only speculate about the long-term success of root coverage in this case. However, evidence suggests that creeping attachment and improvement in root coverage are typically observed in cases where CTG is used for root coverage.^22-26^

## Conclusion

Within the limitations of this study, it might be concluded that even in difficult cases with severe gingival recession with unfavorable prognosis, root coverage could potentially be achieved, at least in the short term using CTG with the tunneling technique.

## Authors’ contributions

AS and RA contributed towards literature search and the construction of the manuscript whereas, Mohammad Ahmad Javaid performed the surgical procedure and provided oversight for the final article structure.

## Competing interests

Authors declare that there is no conflict of interests or competing interests.

## Ethics approval

Ethics approval was obtained through Institutional Ethics Committee.
